# Blood Glucose Prediction Method Based on Particle Swarm Optimization and Model Fusion

**DOI:** 10.3390/diagnostics12123062

**Published:** 2022-12-06

**Authors:** He Xu, Shanjun Bao, Xiaoyu Zhang, Shangdong Liu, Wei Jing, Yimu Ji

**Affiliations:** 1 School of Computer Science, Nanjing University of Posts and Telecommunications, Nanjing 210023, China; 2 Jiangsu High Technology Research Key Laboratory for Wireless Sensor Networks, Nanjing 210023, China; 3 Institute of High-Performance Computing and Big Data, Nanjing University of Posts and Telecommunications, Nanjing 210023, China; 4 Nanjing Center of HPC China, Nanjing 210023, China; 5 Jiangsu Research Engineering of HPC and Intelligent Processing, Nanjing University of Posts and Telecommunications, Nanjing 210023, China; 6 Yuwell Group, Nanjing 210023, China

**Keywords:** diabetes mellitus, blood glucose, particle swarm optimization, model fusion

## Abstract

Blood glucose stability in diabetic patients determines the degree of health, and changes in blood glucose levels are related to the outcome of diabetic patients. Therefore, accurate monitoring of blood glucose has a crucial role in controlling diabetes. Aiming at the problem of high volatility of blood glucose concentration in diabetic patients and the limitations of a single regression prediction model, this paper proposes a method for predicting blood glucose values based on particle swarm optimization and model fusion. First, the Kalman filtering algorithm is used to smooth and reduce the noise of the sensor current signal to reduce the effect of noise on the data. Then, the hyperparameter optimization of Extreme Gradient Boosting (XGBoost) and Light Gradient Boosting Machine (LightGBM) models is performed using particle swarm optimization algorithm. Finally, the XGBoost and LightGBM models are used as the base learner and the Bayesian regression model as the meta-learner, and the stacking model fusion method is used to achieve the prediction of blood glucose values. In order to prove the effectiveness and superiority of the method in this paper, we compared the prediction results of stacking fusion model with other 6 models. The experimental results show that the stacking fusion model proposed in this paper can accurately predict blood glucose values, and the average absolute percentage error of blood glucose prediction is 13.01%, and the prediction error of the stacking fusion model is much lower than that of the other six models. Therefore, the proposed diabetes blood glucose prediction method in this paper has superiority.

## 1. Introduction

Diabetes is a group of metabolic diseases characterized by chronic hyperglycemia. It is caused by various reasons and is often accompanied by a series of metabolic disorders of sugar, protein, fat, water, and electrolytes in the body due to defective insulin secretion or insulin function. According to relevant data and studies, the prevalence of diabetes in China is the highest in the world. Diabetes is difficult to cure and may lead to many complications that seriously threaten the lives of patients [1]. Currently, many experts and scholars have made great contributions to the study of blood glucose value prediction, and many different prediction methods have been proposed. At present, the mainstream blood glucose value prediction methods are mainly divided into two aspects, one is the blood glucose value prediction method based on human physiological model, and the other is the data-driven blood glucose value prediction method.

Physiological model-based methods for predicting blood glucose values are mainly based on medical theory and can be divided into maximum and minimum models. The basic idea of the method of blood glucose prediction based on the extreme value model is to simulate the metabolic process of blood glucose values by covering all metabolic information of the human body to the maximum extent and integrating this information. The minimum model-based method for predicting blood glucose values is relatively simple, as its basic idea is to not include other metabolic information of the human body, and only the core equation is used to represent the relationship between blood glucose values and insulin. In 2021, Wang et al. proposed an Random search and Grid search (RG) hyperparametric optimization method based on the sequential use of stochastic search and grid search for improving the prediction of blood glucose levels from boosted integrated learning models. Based on historical medical data collected through physical examination methods, 40 human health indicators were used to indirectly predict patients’ blood glucose levels. Experiments with real clinical data demonstrate that the proposed RG dual optimization approach helps improve the predictive performance of its rich set of four state-of-the-art Boosting integrated learning models, achieving Mean Squared Error (MSE) improvements from 1.47% to 24.40% and Root Mean Squared Error (RMSE) improvements from 0.75% to 11.54% [2]. In the same year, Yusra. Obeidat et al. used a machine learning (ML) based model to track patients’ glucose levels and predict the appropriate amount of insulin and used an artificial neural network (ANN) model to predict blood glucose. The experimental results showed that the method had a mean square error of 5.79 [3]. Since human physiological metabolism is a complex process, which is affected by many external factors, and physiological modeling requires consideration of individual differences and knowledge of pharmacology, the accuracy of predicting blood glucose values by physiological models is often not guaranteed.

The Continues Glucose Monitoring System (CGMS) is an innovative technological breakthrough in the field of glucose monitoring. It reflects blood glucose levels by continuously monitoring the glucose concentration in the subcutaneous intercellular fluid [4]. Blood glucose homeostasis is an important measure of the health status of diabetic patients, and, as there is no cure for diabetes, it is important for the control and treatment of diabetic patients if the blood glucose level can be predicted quickly and accurately. Based on the data provided by CGMS, researchers have proposed various prediction methods to predict blood glucose values by building data-driven models, such as autoregressive (AR) [5,6,7] models, support vector machines [8,9], and neural networks [10,11]. In 2018, Takoua. Hamdi et al. used support vector machine and differential evolution algorithm to predict blood glucose values in type 1 diabetes, and the experimental results proved that the support vector machine model based on differential evolution algorithm achieved high prediction accuracy [12]. In the same year, Jaouher Ben. Ali et al. proposed a new method based on artificial neural networks for predicting blood glucose levels in type 1 diabetes, and experimental results showed that the method was accurate and adaptive [13]. In 2019, Dong et al. proposed a new Blood glucose (BG) prediction method Clu-RNN (Cluster-Recurrent Neural Networks), which is based on RNN integrating the processing of clustering into classical RNN, and after experiments showed that the method has some improvement in blood glucose prediction accuracy compared with support vector machine and other RNN methods [14]. In 2020, Ganjar Alfian et al. used XGBoost model to predict future blood glucose values in type 1 diabetic patients and experimental results showed that the accuracy of blood glucose prediction based on XGBoost model was better than other models [15]. In the same year, Wang et al. used the more novel LightGBM model and optimized the model parameters using a Bayesian optimization algorithm to finally predict blood glucose levels, and the experiments demonstrated that the optimized model had higher prediction accuracy than the XGBoost and Categorical Boosting (CatBoost) models [16]. 

Through the above study, it is easy to find that in the previous studies on the prediction of blood glucose values, most scholars used a single model. Although these models have good performance, there are more parameters, and the choice of different parameters have some influence on the experimental results. In addition, a single model has its limitations and often does not work as well as it should when dealing with uncertain data. Therefore, fusing different models to take advantage of their respective strengths. The accuracy of blood glucose prediction can be further improved. To solve the above problems, this paper proposes a blood glucose prediction method based on particle swarm optimization and model fusion based on the blood glucose data provided by CGMS. The method mainly optimizes XGBoost and LightGBM models with hyperparameters by particle swarm optimization algorithm, and then improves blood glucose prediction accuracy by using the optimized XGBoost and LightGBM models as base learners and Bayesian regression models as meta-learners for model fusion based on the idea of stacking model fusion. The main innovations of this paper are four points as follows:

(1) The main methods of current research are to learn the training model from the historical blood glucose data of the same patient. This paper is to build the model by learning the historical blood glucose data of different patients, so as to achieve the prediction of blood glucose level of brand-new patients.
(2) The Kalman filter algorithm is used to smooth and reduce the noise of the actual operating current signal of the sensor to reduce the influence of noise on the data and improve the accuracy of the experimental results.
(3) Hyperparameter optimization of XGBoost and LightGBM models uses particle swarm optimization algorithm to achieve their near-optimal performance.
(4) The stacking model fusion method is used to integrate the prediction results of the base learners to take advantage of their respective advantages and effectively reduce the prediction error, so as to improve the accuracy of blood glucose prediction.

## 2. Introduction of Related Algorithms

### 2.1. Particle Swarm Optimization Algorithm

Particle swarm optimization algorithm (PSO) [17,18] is an evolutionary algorithm technique based on the study of bird predation behavior. The basic idea of this algorithm is to find the optimal solution through collaboration and information sharing among individuals in a population.

#### 2.1.1. Fundamentals

In the d-dimensional continuous search space, the *i*-th (*i* = 1, 2, …, *n*) particle in the particle population is defined: $x_{i}^{k}=$($x_{i1}^{k},x_{i2}^{k},\;\ldots ,\;x_{id}^{k}$), denoting the position of the *i*-th particle at moment *k*; the optimal position experienced by each particle is denoted as $p_{i}^{k}=\left(p_{i1}^{k},p_{i2}^{k},\;\ldots ,\;p_{id}^{k}\right)$, and the optimal position experienced by the population is denoted as $p_{g}^{k}=\left(p_{g1}^{k},p_{g2}^{k},\;\ldots ,\;p_{gd}^{k}\right)$. According to the above theory, the velocity $v_{id}^{k+1}$ and position $x_{id}^{k+1}$ of each particle in the population at the moment *k*+1 is updated as shown below.

(1) $$v_{in}^{k+1}=w^{k}v_{id}^{k}+c_{1}r_{1}\left(p_{id}^{k}-x_{id}^{k}\right)+c_{2}r_{2}\left(p_{gd}^{k}-x_{id}^{k}\right)$$

(2) $$x_{id}^{k+1}=x_{id}^{k}+v_{id}^{k+1}$$

where *w* denotes the inertia weight, *k* denotes the number of current iterations, $r_{1}$ and $r_{2}$ are random numbers between (0, 1), and $c_{1}$ and $c_{2}$ are acceleration factors, which are individual learning factors and group learning factors, respectively.

#### 2.1.2. Algorithm Pseudocode

The particle swarm optimization Algorithm 1 (PSO) pseudocode is as follows:
**Algorithm 1:** Particle swarm optimization algorithm (PSO)Input:  Position *x*, velocity *v* of each particle  Dimension *d* of the particle, learning factor $c_{1}$$,\;c_{2}$
Output: the optimal position of the particle *x*1:   FOR each particle *i*   // Iterate over each particle2:     FOR each particle *i*   // Iterate over each particle3:       Initialize position $x_{id}$ randomly within peimissible range // Initialize particle position4:       Initialize velocity $v_{id}$ randomly within peimissible range // Initialize particle velocity5:     END FOR6:   END FOR7:   Iteration *k* = 100 // Set the number of iterations8:   Do9:     FOR each particle *i*10:       Calculate fitness value // Calculate the particle fitness (fitness function is: MAPE)11:       IF the fitness value is better than $p_{id,pbest}^{k}$ in history // Optimal particle position12:         Set current fitness value as the $p_{id,pbest}^{k}$
13:       END IF14:     END FOR15:   Choose the particle having the best fitness value as the $p_{d,gbest}^{k}$ // Group optimal position16:     FOR each particle *i*17:       FOR each dimension *d*18:         Calculate velocity according to the equation19:         $v_{id}^{k+1}=\omega v_{id}^{k}+c_{1}r_{1}\left(p_{id,pbest}^{k}-x_{id}^{k}\right)+c_{2}r_{2}\left(p_{d,gbest}^{k}-x_{id}^{k}\right)$ //Update speed20:         Update particle position according to the equation21:         $x_{id}^{k+1}=x_{id}^{k}+v_{id}^{k+1}$ // Update the position of the particle22:       END FOR23:     END FOR24:   *k* = *k* + 125:   WHILE maximum iterations or minimum error criteria are not attained // Termination Conditions

### 2.2. The Stacking Model Framework

**Figure 1:**
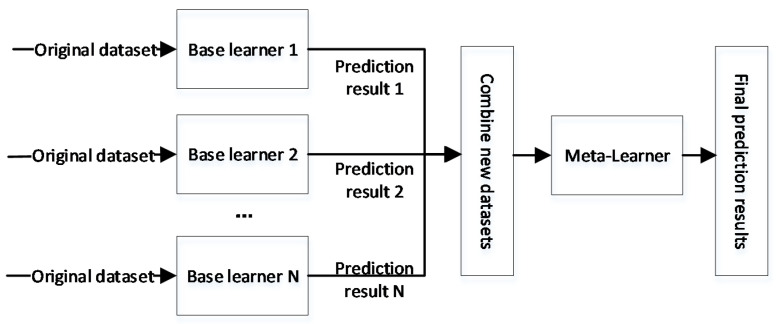
Stacking integrated learning approach.

The basic idea of stacking integrated learning is that multiple base learners are trained using the original dataset, and the prediction output of each base learner is fed into the meta-learner as a new dataset, and the output of the meta-learner is used as the final prediction [19,20,21], as shown in Figure 1.

In which the K-fold (K = 5) cross-validation method is used in fusing N (N ≥ 1) base learners and 1 meta-learner. The exact flow of the algorithm is as follows:

(1) Divide the data set into training set and test set, randomly divide the training set into K copies by non-repetitive sampling and select K − 1 copies of them each time as the training set of the base learner and 1 copy as the validation set.
(2) Repeating the operation K times, with each subset having a chance to become a validation set.
(3) Predicting the validation set (1 copy) with the base learner model obtained by training each training set (K − 1 copies).
(4) Step 3 loops K times to obtain K base learner models and a new column of data of the same length as the training set (a combination of the predictions from the validation set).
(5) With N base learners, step 4 loops N times to obtain N new columns of data as the training set for the meta-learner.
(6) The K models obtained in step 4 are predicted for the test set and the predictions have k columns, the arithmetic mean of which is taken after 1 column.
(7) With N base learners, step 6 loops N times to obtain N new columns of data as the test set for the meta-learner.
(8) The trained meta-learner using the training set predicts the test set and the result is the final prediction result.

**Figure 2:**
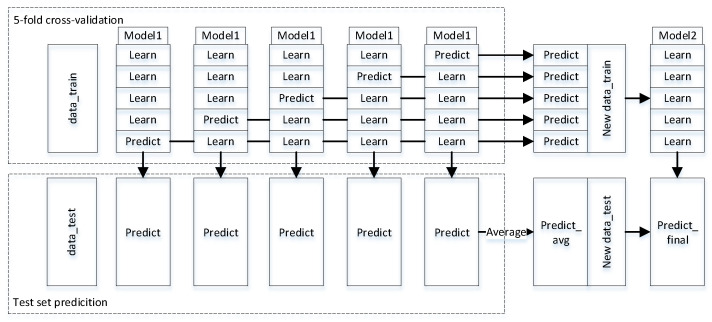
Principle diagram of stacking model fusion.

To facilitate the elaboration of the principle. The principle of choosing 1 base learner and 1 meta learner to do stacking model fusion is shown in Figure 2.

## 3. Construction of the Fusion Model Based on PSO

### 3.1. Data Source

The data used in this paper are from the sensor data provided by Yuwell Group, which contains the blood glucose data of 33 patients on 24 May and the blood glucose data of 10 patients on 16 June, totaling 464,438 and 113,591 items, respectively. Among them, the data on 24 May were used as the training set of the model and the data on 16 June were used as the test set of the model, in which only blood glucose concentration values containing fingertip blood collection measurements, (i.e., actual blood glucose values), totaling 1281 entries, were used to judge the model prediction results. The main data items of the data are shown in Table 1.

### 3.2. Data Pre-Processing

#### 3.2.1. Feature Selection

**Figure 3:**
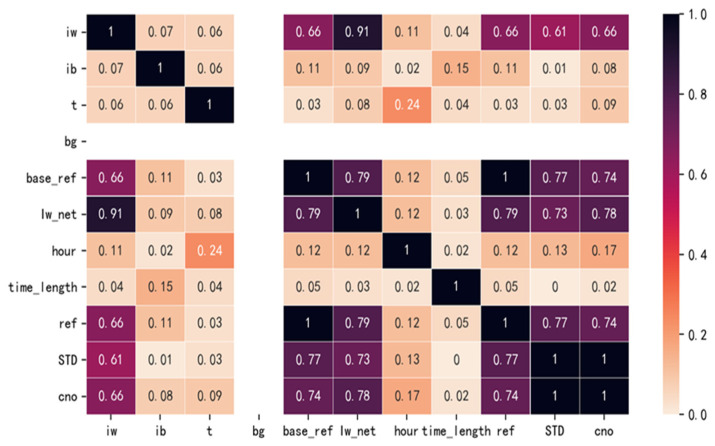
Heat map of feature-dependent sparse matrix.

The main data items in the dataset of this paper include sensor running time, operating current signal value, blank current signal value, body surface temperature, actual blood glucose value, calibration value, Continues Glucose Monitoring (CGM) concentration, actual current value involved in blood glucose calculation, blood glucose concentration value generated by the Kellett algorithm without calibration, and blood glucose value after calibration based on the most recent fingertip blood collection value. In order to better select the features, this paper uses the heat map method to show the correlation between each feature and the real blood glucose value. In addition, to augment the features, the current operating hour value of the sensor (hour) and the sensor operating hour (in minutes) (time_length) were added as new features after analysis. Figure 3 shows the heat map of the characteristic correlation coefficient matrix, the higher the value the higher the correlation.

From Figure 3, it can be seen that bg, cno, and other features are not correlated. Additionally, base_ref, ref, and STD are more correlated with cno, but it is known from Section 2.1 that they are the output and calibration values of the Fisheye Kellett algorithm and cannot be used as features. Furthermore, iw, ib, t, Iw_net, and cno have either high or low correlation, and the newly added features hour, time_length, and cno also have some correlation and can be used as features. Therefore, the final features selected in this paper are: iw, ib, t, Iw_net, hour, and time_length.

#### 3.2.2. Kalman Filtering Algorithm

Among the many blood glucose data acquired by the CGMS system, the current signal from the sensor has certain burr and noise, which will affect the model effect if the current signal is directly input to the model. Therefore, the original data needs to be smoothed and noise reduced.

Kalman filter (KF) [22] algorithm, is a recursive predictive filtering algorithm. It provides an efficient and computable method for estimating the process state and minimizing the mean square error of the estimate. The Kalman filter is widely used and powerful as it can estimate the past and current state of the signal, and even the future state, making it ideal to use Kalman filtering in a system, like the one used in this paper, where the sensor signal varies continuously. It can be specifically divided into two steps, the prediction process and the correction process [23].

(1). Prediction process: the state of the next moment is estimated by the state of the previous moment, and the calculation formula is as follows:

(3) $$x_{k}=Ax_{k-1}+Bu_{k}$$

(4) $$P_{k}=AP_{k-1}A^{T}+Q$$

where $x_{k}$ and $u_{k}$ denote the prior estimates and system control quantities, respectively, *A* and *B* are the state transfer matrix and control matrix, respectively, and *P* and *Q* are the error covariance and process noise, respectively.

(2). Correction process: according to the current moment of the state estimate and the observed state, the optimal state is estimated, and the calculation formula is as follows:

(5) $$K_{k}=\frac{P_{k}H^{T}}{HP_{k}H^{T}+R}$$

(6) $${\hat{x}}_{k}=x_{k}+K_{k}\left(Z_{k}-Hx_{k}\right)$$

(7) $$P_{k}=\left(1-K_{k}H\right)P_{k}$$

where $K_{k}$ is the Kalman gain, *R* is the noise average of the sensor, *H* is the state variable of the transformation matrix,$\;Z_{k}$ is the measured value of the sensor, and ${\hat{x}}_{k}$ is the posterior estimate, i.e., the current optimal solution.

**Figure 4:**
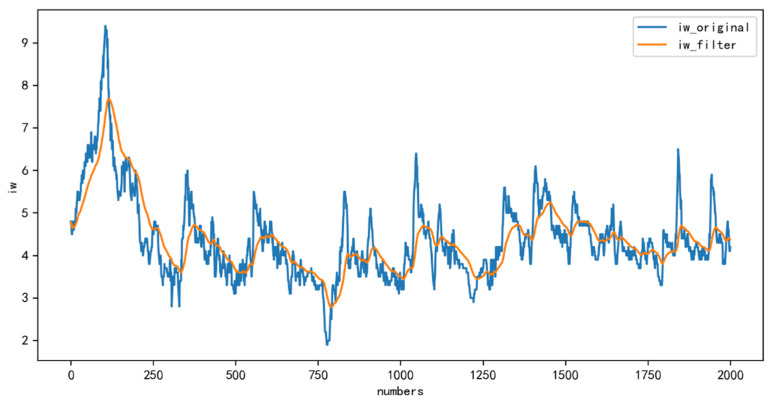
Comparison of the original values of training set iw with the filtered values.

Taking the training set as an example, the iw values of the training set are smoothed and noise-reduced by the Kalman filtering algorithm, and the comparison results before and after noise reduction are shown in Figure 4.

#### 3.2.3. Data Normalization

Data normalization is the scaling of data to a smaller specific range. Data normalization can speed up the computation of data and may even improve the prediction accuracy of the model. Common methods of data normalization include: min-max normalization [24], log function conversion, atan function conversion, *z*-score normalization [25], and fuzzy quantification method. Since the fluctuation range of patients’ blood glucose values in the dataset is large and there are outliers, the *z*-score method is chosen to normalize all data in this paper, and the transformation function is.

(8) $$z=\frac{x-\mu }{\sigma }$$

where *μ* is the mean, *σ* is the standard deviation, and *x* and *z* are the data before and after normalization.

### 3.3. Particle Swarm Optimization Base Learner

The data of 27 patients were selected from the blood glucose data of 33 patients on 24 May for model training and parameter optimization, and the data of the remaining 6 patients containing real blood glucose values were used as the test set to evaluate the model of tuning parameters.

In the process of hyperparameter optimization of the model, we used the particle swarm optimization algorithm, the average absolute percentage error of the model is first selected as the fitness function of the particles. Next, the particle swarm parameters are initialized, the position and velocity of each particle are randomly initialized, then the fitness of each particle is calculated. Finally, we determined whether the location of each particle is the current optimal value based on the adaptation size. If it is the optimal value, the value of the original position is replaced, and then the position and velocity of each particle are updated. When the set maximum number of iterations is reached, the position of the particle with the current optimal value is output. It is worth noting here that the position of the particle represents the value of the model parameters.

In the process of using the optimization model for the prediction of the test set, the model parameters as the velocity update equation for the particles is $v_{id}^{k+1}=\omega v_{id}^{k}+c_{1}r_{1}\left(p_{id,pbest}^{k}-x_{id}^{k}\right)+c_{2}r_{2}\left(p_{d,gbest}^{k}-x_{id}^{k}\right)$, the update formula for the position is $x_{id}^{k+1}=x_{id}^{k}+v_{id}^{k+1}$. Where the total number of particles N is 10, the particle dimension *d* is 2, the number of iterations *k* is 100, and the inertia weight *w* is 0.5 and the learning factors *c*_1_ and *c*_2_ are set to 2. Table 2 shows the main parameters and descriptions of the XGBoost and LightGBM models.

The range of values of the parameters position and speed of XGBoost and LightGBM are shown in Table 3. 

### 3.4. Stacking Model Fusion

Stacking is a rather special hierarchical structure, where the first layer is a different base learner and the second layer above the base learner output results in a meta-learner for the input data. In order to prevent overfitting, based on stacking model fusion, this paper proposes to add a five-fold cross-validation with two repetitions in the meta-learner training process to avoid the overfitting problem.

**Figure 5:**
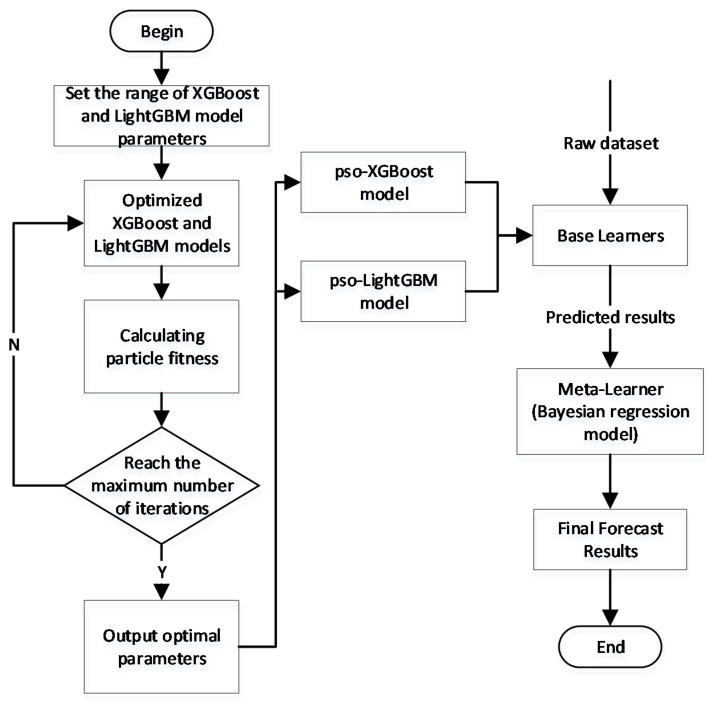
Flow chart of the construction of the fusion model based on particle swarm optimization.

In order to improve the accuracy of blood glucose prediction, combining the advantages of strong generalization ability and high operational efficiency of XGBoost and LightGBM models, this paper proposes to construct a blood glucose prediction model based on particle swarm optimization and model fusion, using XGBoost model and LightGBM model after particle swarm optimization in the first layer and weak learner-Bayesian regression model in the second layer. The specific flow chart of the construction of the fusion model based on particle swarm optimization is shown in Figure 5.

## 4. Experimental Results and Analysis

### 4.1. Experimental Environment

The computer configuration used in this paper is Intel i5-7300 2.5GHz quad-core quad-thread CPU, 16G RAM, Windows 10 operating system, programming language python, programming environment pycharm, and libraries, such as numpy, sklearn, pandas, xgboost, lightgbm are used, respectively.

### 4.2. Model Evaluation Metrics

In this paper, two evaluation metrics, mean absolute percentage error (MAPE) and root mean square error (RMSE), are used to evaluate the performance of the model. Mean absolute percentage error (MAPE) is one of the most popular metrics used to evaluate the prediction performance, and root mean square error (RMSE) is usually used as a measure of the prediction results of machine learning models, and their smaller values indicate a higher accuracy of blood glucose value prediction. Their specific evaluation functions are formulated as follows:

(9) $$\mathrm{MAPE}=\frac{1}{N}\sum_{i=1}^{N}\frac{\left|{\hat{y}}_{i}-y_{i}\right|}{y_{i}}\times 100\%$$

(10) $$\mathrm{RMSE}=\sqrt{\frac{1}{N}\sum_{i=1}^{N}{\left({\hat{y}}_{i}-y_{i}\right)}^{2}}$$

where *N* is the number of samples, ${\hat{y}}_{i}$ is the predicted blood glucose value, and $y_{i}$ is the true blood glucose value.

### 4.3. Analysis and Comparison of Filtering Results

In this paper, the use of Kalman filtering algorithm is able to reduce the influence of sensor signal noise on blood glucose prediction results, as shown in Table 4.

As can be seen from Table 4, the prediction error of blood glucose was significantly reduced after smoothing and noise reduction in the original data using the Kalman filtering algorithm. In terms of the MAPE metric, the prediction error of KF-XGBoost is 1.23% lower than that of XGBoost, and the prediction error of KF-LightGBM is 1.04% lower than that of LightGBM. In terms of RMSE metrics, the prediction error of KF-XGBoost is 3.12% lower than that of XGBoost, and the prediction error of KF-LightGBM is 1.7% lower than that of LightGBM. It can be seen that the Kalman filter algorithm can significantly improve the prediction accuracy of blood glucose after processing the raw data.

### 4.4. Analysis and Comparison of Optimization Results

**Figure 6:**
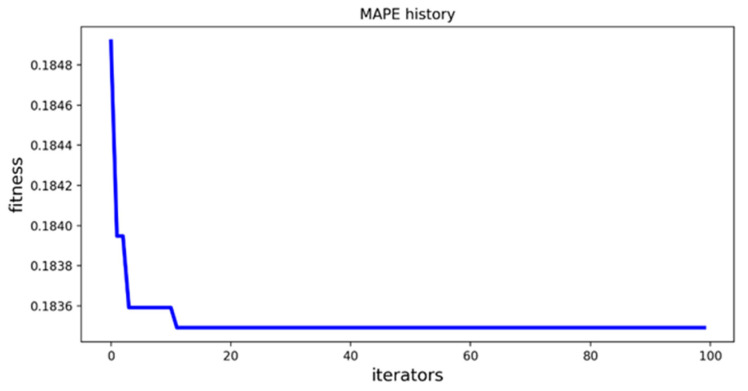
PSO-XGBoost parameter iteration curve.

**Figure 7:**
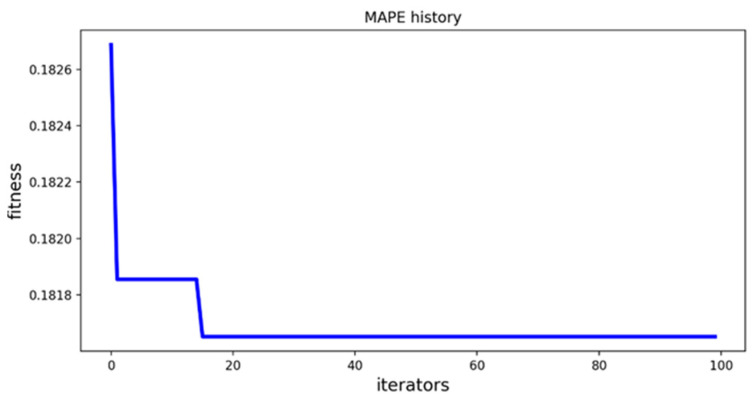
PSO-LightGBM parameter iteration curve.

Figure 6 shows the curve of the fitness value with the number of PSO iterations when the particle swarm optimization algorithm optimizes the parameters of XGBoost model, and Figure 7 shows the curve of the fitness value with the number of PSO iterations when the particle swarm optimization algorithm optimizes the parameters of LightGBM model. From Figure 6 and Figure 7, it can be seen that the optimal combination of parameters for the XGBoost model can be searched when the number of iterations reaches about 10, and the optimal combination of parameters for the LightGBM model can be searched when the number of iterations reaches about 20.

The optimization results using PSO with the set termination iteration conditions are shown in Table 5.

Based on the dataset, the model was trained using the optimized parameters, and the prediction results are shown in Table 6.

As it can be seen in Table 6, the prediction error of blood glucose was significantly reduced after hyperparameter optimization of the model using the particle swarm optimization algorithm. In terms of the MAPE metric, the prediction error of PSO-XGBoost is 0.92% lower than that of XGBoost, and the prediction error of PSO-LightGBM is 1.01% lower than that of LightGBM. In terms of RMSE metrics, the prediction error of PSO-XGBoost is 1.53% lower than that of XGBoost, and the prediction error of PSO-LightGBM is 4.54% lower than that of LightGBM. It can be seen that the particle swarm optimization algorithm can significantly improve the blood glucose prediction accuracy after hyperparameter optimization of the model.

### 4.5. Analysis and Comparison of Model Fusion Results

In this paper, the output results of the first layer XGBoost and LightGBM models are input into the second layer Bayesian regression model as a way to predict blood glucose values, and the prediction results are shown in Table 7. In order to better verify the effect of the models, this paper compares XGBoost, LightGBM, CatBoost, Bayesian regression, linear regression, and Kellett algorithm, and evaluates the effect of each model by calculating the mean absolute percentage error and root mean square error.

As can be seen from the table, compared with the base learner, CatBoost, linear regression, Bayesian regression and Kellett algorithm, the blood glucose prediction method based on particle swarm optimization and model fusion proposed in this paper has a great advantage in prediction performance. In terms of MAPE metrics, the prediction effect improves by 1.76% and 2.07% over the base learner and 5.04% over the Kellett algorithm. In the RMSE metric, the prediction effect improves 1.29% and 4.98% over the base learner, and 4.77% over the Kellett algorithm. In terms of both metrics, the blood glucose prediction method proposed in this paper is much better than CatBoost, linear regression, Bayesian regression, and Kellett algorithm in terms of prediction effectiveness and generalization ability.

**Figure 8:**
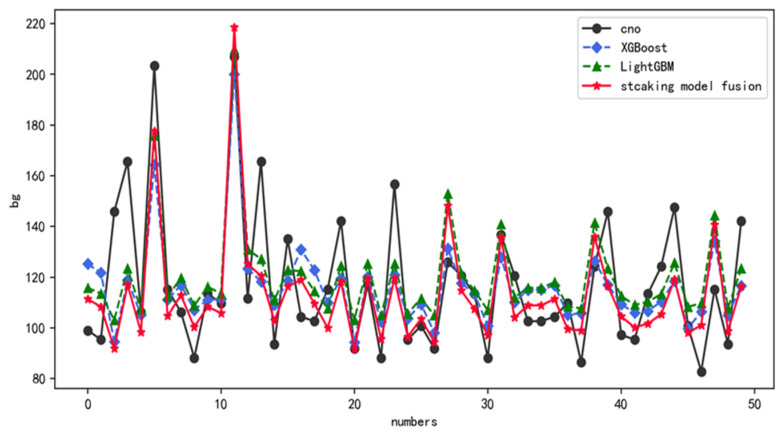
Comparison of prediction results of base learner and fusion model.

Figure 8 shows the prediction results of the base learner and the fusion method on the patient’s blood glucose values, and from Figure 8, it can be seen that the fusion method proposed in this paper has the best prediction results, the LightGBM model has the worst prediction results, and the XGBoost model has the prediction results in between. Combined with the evaluation indexes in Table 7, the MAPE of the fusion method is 13.01% and the RMSE is 23.15, the MAPE of the XGBoost model is 14.77% and the RMSE is 24.44, and the MAPE of the LightGBM model is 15.08% and the RMSE is 26.08. The values of MAPE and RMSE of the fusion method proposed in this paper are the smallest, and then the highest prediction accuracy of the fusion method, which is consistent with the prediction results in Figure 8.

**Figure 9:**
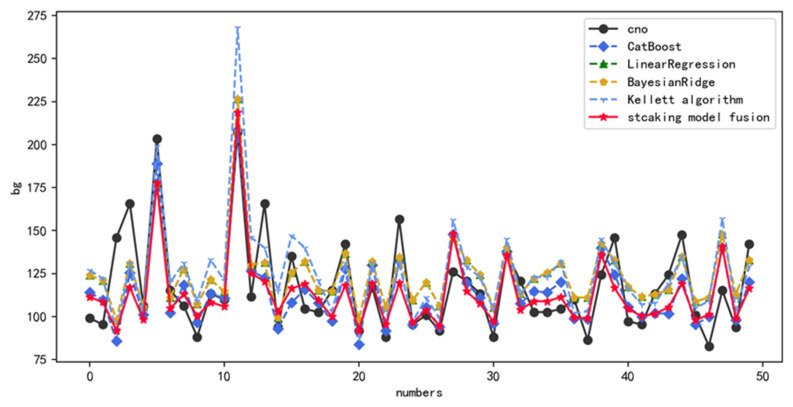
Comparison of model prediction results.

Figure 9 shows the prediction results of CatBoost, linear regression, Bayesian regression, Kellett algorithm, and fusion methods on patients’ blood glucose values. From Figure 9, it can be seen that the fusion method proposed in this paper has the best prediction results, the CatBoost model has the second-best prediction effect, Kellett algorithm has the worst prediction effect, and linear regression and Bayesian regression have prediction results between them. Combined with the evaluation indexes in Table 7, the MAPE of the fusion method is 13.01% and the RMSE is 23.15, the MAPE of the CatBoost model is 14.44% and the RMSE is 24.35, the MAPE of the Kellett algorithm is 18.05% and the RMSE is 27.92, and the MAPE of linear regression with Bayesian regression is 15.09% and the RMSE is 24.08, which is consistent with the predicted results in Figure 9.

In general, Figure 8 and Figure 9 show the prediction results of each model for the first 50 bars of patients’ blood glucose in the test set compared with the true blood glucose values. From the Figure 8 and Figure 9, it can be seen that the prediction results of the stacking fusion model are very close to the true blood glucose values in most cases compared with XGBoost, LightGBM, CatBoost, Bayesian regression, linear regression, and Kellett algorithm, which have a better fit and prediction results. It can be seen that the blood glucose prediction method based on particle swarm optimization and fusion model proposed in this paper has certain superiority.

**Figure 10:**
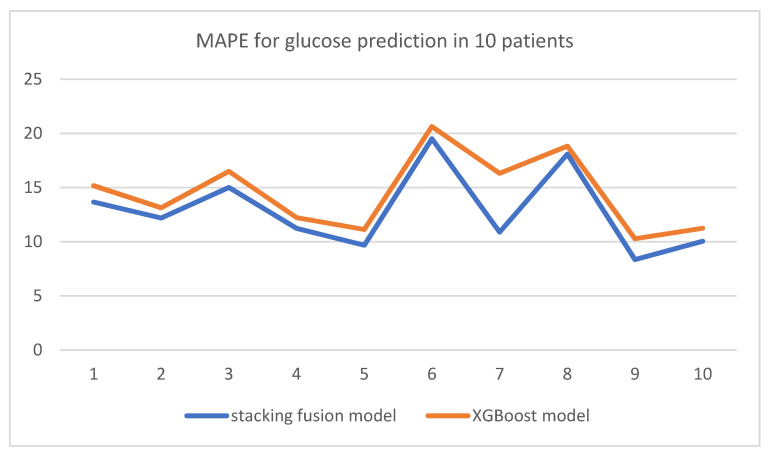
MAPE for glucose prediction in 10 patients.

From the results in Table 7, the prediction error of the XGBoost model is closer to that of the prediction method in this paper. In order to better show the comparison results, the prediction results are broken down by patients below. Table 8 and Figure 10 show the comparison results of the mean absolute percentage error of blood glucose prediction for 10 patients between the particle swarm optimization and model fusion-based blood glucose prediction method proposed in this paper and the XGBoost model.

As can be seen from Table 8 and Figure 10, the method proposed in this paper outperformed the XGBoost model in terms of blood glucose prediction for all 10 patients, with errors between 1% and 2% lower than the XGBoost model. Especially for the blood glucose prediction of patient No. 7, the two pulled apart a large gap. This shows that the method proposed in this paper is more accurate than the XGBoost model in prediction, has better generalization ability, and has certain superiority in predicting the blood glucose level of brand-new patients.

## 5. Discussion

The current signal of the sensor obtained from CGMS has a certain burr and noise, and this paper uses the Kalman filtering algorithm to attempt to reduce the influence of sensor signal noise on the blood glucose prediction results. Through the experimental results, it is found that the filtered data is closer to the real current signal of human body, which has a significant improvement on the prediction accuracy of blood glucose value. The XGBoost and LightGBM models themselves have a large number of parameters, and different parameter pairings have a certain impact on the experimental results. In this paper, the particle swarm optimization algorithm is used to optimize the hyperparameters of the XGBoost and LightGBM models to achieve the best prediction results. The experimental results show that the prediction accuracy of the optimized model has been improved compared with that before the optimization. In addition, in the process of blood glucose prediction, a single model has certain limitations and often does not work as well as it should in dealing with uncertain data. The stacking model fusion method can fuse different models and bring their respective advantages into play to further improve the accuracy of blood glucose prediction. Through the experimental results, it was found that the fused model has further improvement in blood glucose prediction accuracy than the original model. However, this paper aimed to achieve the prediction of blood glucose levels in brand new patients, which has some value for practical clinical application and deserves to be studied in depth.

## 6. Summary

In this paper, the proposed method of blood glucose prediction based on particle swarm optimization and fusion model firstly uses the Kalman filter algorithm and *z*-score method to preprocess the sensor data, then uses particle swarm optimization algorithm to optimize the hyperparameters of XGBoost and LightGBM models, and finally uses the optimized XGBoost and LightGBM models as the base learner and Bayesian regression models are used as meta-learners, and the models are trained and tested using sensor data to finally achieve more accurate prediction of blood glucose values. Through experimental comparison, the prediction effect of the fusion model based on particle swarm optimization is significantly better than the base learner, with 1.76% and 2.07% improvement in the prediction accuracy, and has higher prediction accuracy compared with other regression models.

The shortcoming of this study is that the best combination of models is selected manually rather than through an intelligent combination mechanism, so its efficiency is not high. It is hoped that in future research a base learner library can be established and combined with different swarm intelligence optimization algorithms to achieve the automatic combination of base learners for optimal prediction.

## Figures and Tables

**Table 1:** Main data items.

Data Items	Description
itime	Running time
iw	Operating current
ib	Blank current
t	Body surface temperature
cno	Actual blood glucose value
bg	Sensor calibration value
base_ref	CGM concentration
Iw_net	Actual operating current
ref	Uncalibrated blood glucose value
STD	Calibrated blood glucose value

itime in the table refers to the current running time of the sensor, every three minutes; cno is the actual blood glucose concentration obtained by fingertip blood collection; bg is the calibration value, and when it is not 0, it means that the bg value calibrates CGM at this time, base_ref is the blood glucose value generated by the Kellett algorithm; STD is the blood glucose value after calibration based on the last fingertip blood collection.

**Table 2:** Main parameters and descriptions of XGBoost and LightGBM models.

Parameter	Name	Meaning Set Value
max_depth	Maximum depth of the tree	3
reg_lambda	L2 Regularization term weights	1
learning_rate	Learning Rate	0.1
n_estimators	Number of iterations	100
num_leaves	Number of leaf nodes of the tree	20

**Table 3:** Optimization scheme of XGBoost and LightGBM model parameters.

Parameter	Range of Values	Speed Range
reg_lambda(XGBoost)	[2, 8]	[0, 1]
n_estimators(XGBoost)	[50, 100]	[0, 10]
num_leaves(LightGBM)	[2, 15]	[0, 1]
n_estimators(LightGBM)	[1, 20]	[0, 1]

**Table 4:** KF filtering result.

Model	MAPE (%)	RMSE
XGBoost	16.00	27.56
KF-XGBoost	14.77	24.44
LightGBM	16.12	27.78
KF-LightGBM	15.08	26.08

**Table 5:** PSO optimization results.

Parameter	Final Value
reg_lambda(XGBoost)	4
n_estimators(XGBoost)	52
num_leaves(LightGBM)	14
n_estimators(LightGBM)	20

**Table 6:** Comparison of PSO optimization results.

Model	MAPE (%)	RMSE
XGBoost	14.77	24.44
PSO-XGBoost	13.85	22.91
LightGBM	15.08	26.08
PSO-LightGBM	14.07	21.54

**Table 7:** Comparison of the prediction results of each model.

Model	MAPE (%)	RMSE
XGBoost	14.77	24.44
LightGBM	15.08	26.08
CatBoost	14.44	24.35
Linear regression	15.09	24.08
Bayesian regression	15.09	24.08
Kellett algorithm	18.05	27.92
Stacking fusion model	13.01	23.15

**Table 8:** Predicted blood glucose results in 10 patients.

Patient Number	Stacking Fusion Model	XGBoost Model
1	13.66	15.17
2	12.19	13.13
3	15.01	16.50
4	11.23	12.22
5	9.68	11.13
6	19.51	20.64
7	10.89	16.31
8	18.09	18.83
9	8.35	10.28
10	10.04	11.24

## Data Availability

The data that support the findings of this study are available from the corresponding author upon reasonable request.
